# Impact of *KCNJ11* rs5219, *UCP2* rs659366, and *MTHFR* rs1801133 Polymorphisms on Type 2 Diabetes: A Cross-Sectional Study

**DOI:** 10.1900/RDS.2021.17.21

**Published:** 2021-08-01

**Authors:** Irina Alexandrovna Lapik, Rajesh Ranjit, Alexey Vladimirovich Galchenko

**Affiliations:** 1 Department of Preventive and Rehabilitative Dietetics, Federal Research Centre of Nutrition, Biotechnology and Food Safety, Moscow, Russian Federation,; 2 Department of Oncology, Radiology and Nuclear Medicine, Peoples’ Friendship University of Russia, Moscow, Russian Federation,; 3 Department of Medical Elementology, Peoples’ Friendship University of Russia, Moscow, Russian Federation.

**Keywords:** diabetes mellitus, genes, homocysteine, B vitamins, oxidation substrates, carbohydrates, lipids metabolism

## Abstract

**OBJECTIVE:**

Type 2 diabetes (T2D) is a multifactorial disease. Its occurrence and prognosis are affected by many genes, including *KCNJ11, UCP2, and MTHFR*. The objective of this study was to investigate the distribution of various variants of these genes and evaluate their contribution to the outcome of T2D.

**METHODS:**

80 females with T2D and class I-II obesity in the age of 40-65 years old underwent a genetic study, a biochemical blood test, and indirect calorimetry.

**RESULTS:**

Carriers of C/T and T/T genotypes of the *MTHFR* gene had higher levels of cholesterol and triglycerides and lower levels of vitamin B6 and folate. The T/T genotype of the *UCP2* gene was associated with higher levels of glycated hemoglobin, pre- and postprandial glycemia and lipid oxidation rate, lower carbohydrate oxidation, and lower serum vitamin C levels.

**CONCLUSIONS:**

Genotyping *UCP2* and probably *KCNJ11* may help to select the optimal antidiabetic therapy and improve disease prognosis, whereas the *MTHFR* gene may determine the need to monitor group B vitamin status and the risk of dyslipidemia.

## 1 Introduction

Diabetes is one of the leading causes of mortality and morbidity, reaching epidemic dimensions worldwide as approximately 422 million adults are diagnosed with diabetes [1]. A major determining factor of type 2 diabetes (T2D) is obesity. Obesity is already one of the most important medical and social problems in the world. Over the past three decades, the prevalence of overweight and obesity in the world has grown by almost 30-50% [2]. According to the WHO, obesity is currently considered as the most important risk factor for cardiovascular diseases and T2D. Overweight and obesity influence the development of T2D by 44-57%, coronary heart disease by 17-23%, arterial hypertension by 17%, gallstone disease by 30%, osteoarthritis by 14%, and malignant neoplasms by 11% [3], and are even considered to be risk factors for reproductive dysfunction and cancerous diseases [4, 5].

Disorders of carbohydrate metabolism occur in more than half of obese patients [6]. T2D develops because of poor glycemic control in patients with obesity. Medical, social, and economic factors are associated with its steadily increasing prevalence in combination with a high frequency of severe complications [7-9].

A multitude of genes is involved in the progression of T2D. Even more genes affect the development of diabetes complications. Polymorphism in potassium inwardly rectifying channel subfamily J member 11 (*KCNJ11*), uncoupling protein 2 (*UCP2*) and methylenetetrahydrofolate reductase (*MTHFR*) genes may have detrimental effects in patients with T2D. Testing for polymorphisms of *KCNJ11, UCP2*, and *MTHFR*, which are directly involved in the metabolism of glucose, homocysteine (Hcy), and insulin synthesis, is fundamentally important for predicting the risk of T2D and its vascular complications and their prevention [10-13].

The *UCP2* gene is located within chromosome 11q13 [14] and plays an important role in the pathophysiology of T2D because of its proton-moving activity. *UCP2* is a protein that dissociates oxidative phosphorylation. The protein belongs to a family of mitochondrial transport proteins, and it is expressed in adipose tissue and pancreatic islets. By uncoupling β-cell metabolism and ATP production, *UCP2* is involved in the regulation of glucose-stimulated insulin secretion [10]. Meta-analyses from 2011 and 2013 show that a polymorphism in *UCP2* rs659366 increases the susceptibility to T2D in Asians [12, 15]. However, another meta-analysis published in 2013 has shown just the opposite [16]. Furthermore, yet another meta-analysis from 2011 has concluded that the polymorphism is unlikely to be associated with T2D in Asian and European populations [15].

The *KCNJ11* gene (ATP-dependent potassium channel) is located on chromosome 11p15 and plays a vital role in the pathogenesis of T2D. The gene synthesizes a protein, Kir6.2, which is involved in the formation of an ATP-dependent channel that regulates the flow of potassium ions through the cell membrane and eventually links glucose metabolism to the electrical potential of β-cells. The closure of the channel is necessary for the secretion of glucose-stimulated insulin by pancreatic β-cells. The opening of the ATP-dependent channel inhibits insulin secretion. Mutations in the *KCNJ11* gene may cause changes in the structure of the Kir6.2 protein and impair the functioning of the channel, which increases its activity and contributes to the development of insulin resistance. Recent studies have found that the polymorphic marker rs5219 of the *KCNJ11* gene is associated with a risk of developing T2D [13, 17]. A 2014 meta-analysis showed that *KCNJ11* E23K polymorphism (rs5219) was significantly associated with increased T2D risk [18]. However, a study published in 2017 could not confirm an association between T2D and rs5219 variations [19].

Hyperhomocysteinemia (HHcy) is frequently observed in patients with T2D [20]. It was found that Hcy levels in blood plasma significantly correlate with patients’ age , gender, duration of diabetes, and blood pressure. Increased serum Hcy has a damaging effect on the vascular wall caused by oxidative stress. During this condition, there is an increase in the intensity of lipid peroxidation. It is believed that in T2D, HHcy can aggravate endothelial dysfunction, accelerate the development of atherosclerotic processes, and increase the aggregation of platelets and their adhesive properties. As a result, high Hcy concentrations in patients with T2D contribute to the development of micro- and macroangiopathies and hypertension [21].

An increase in serum Hcy levels is frequently associated with a genetically determined deficiency in *MTHFR*, which is involved in the metabolism of this amino acid. As a result of a point mutation, cytosine at position 677 changes to thymine, which is associated with an interruption of folate metabolism and a slowdown of the formation of 5-methylenetetrahydrofolate [22]. The process delivers a methyl group for methylation of Hcy and leads to the development of HHcy. In the presence of a mutant T allele, a thermolabile enzyme appears with a significantly reduced functional activity, which contributes to an increase in the concentration of Hcy in serum [23]. In a number of studies conducted in T2D patients, the association of rs1801133 polymorphism of the *MTHFR* gene with hypertension and ischemic heart disease was detected [11, 24]. Furthermore, the meta-analyses in 2014 and 2019 confirm that the polymorphism in the *MTHFR* gene seems to be associated with T2D [25, 26]. Similarly, individuals with the C/C genotype of the C677T polymorphism were consistently found to be associated with decreased levels of vitamins B6, B9, B12 and increased Hcy levels [27, 28].

To date, there are few studies that have investigated the relationship of polymorphisms of these genes with parameters like vitamins, Hcy, and glycated hemoglobin [29-31]. Hustad *et al*. found an association of vitamin B6 serum levels with different polymorphisms of the *MTHFR* gene [32]. Current studies have not clarified if *KCNJ11* rs5219 and *UCP2* rs659366 polymorphisms affect T2D.

The authors of this study do not harbor illusions about clarifying definitely the effects of *MTHFR* rs1801133, *KCNJ11* rs5219 and *UCP2* rs659366 polymorphisms on the course and prognosis of diabetes. However, because of the current lack of information, the main purpose of this study was to add new information to the general pool of knowledge on this issue. In this study, our intention was to verify possible associations between the variants of *MTHFR, KCNJ11*, and *UCP2* and indicators of the course of T2D, including pre- and postprandial glycemia, indicators of lipidemia, the rate of metabolic oxidation of biosubstrates, and serum concentrations of homocysteine and vitamins B6, B9, and B12, whose deficiency plays one of the key roles in the development of diabetic polyneuropathy.

## 2 Methods

### 2.1 Study design

This research was designed as a cross-sectional observational study, and it was carried out in the Clinic of the Federal State Budgetary Institution of Science, “Federal Research Centre of Nutrition, Biotechnology and Food Safety”. The study included 182 females with T2D and class I-II obesity aged 40 to 65 years old. All patients were tested for polymorphisms in *UCP2, KCNJ11*, and *MTHFR* genes.

### 2.2 Patient sampling

All subjects took the same drug therapy, namely metformin at a dose of 1000 mg/day. Exclusion criteria were: the presence of severe cardiovascular, neurological, infectious, or dermatological complications, insulin therapy, or another glucose-controlling therapy different from those described above. During the course of the study, 102 subjects were excluded from the experiment because they required a change in antihyperglycemic therapy or additional therapy because concomitant diseases were detected. Eventually, 80 people were included in the study.

This procedure for sample selection was applied to maximize sample homogeneity and to reduce the likelihood of bias. Age and sex parameters were chosen to obtain a group with maximum homogeneity and without serious complications or comorbidities. Furthermore, this group had a relatively low risk of being excluded from the study as they were not at risk of a change in hypoglycemic therapy. The majority of these uncomplicated patients attending the clinic received metformin 1000 mg/day therapy. Therefore, the study was standardized exactly according to this scheme of pharmacotherapy. All patients were informed about the experiment and provided written consent for participating in the study. The study was approved by the Ethical Committee of the Federal Research Centre for Nutrition, Biotechnology and Food Safety (protocol No. 17 from 10.24.2012).

### 2.3 DNA extraction and analysis

To reduce the risk of systemic error, all series of experiments were performed on the same analyzers, using reagents of the same series. All DNA samples were extracted from the patients’ blood by standard methods using reagent “RealBest DNA Extraction 3” (Novosibirsk, Russia). The study of polymorphisms in rs5219 of the *KCNJ11* gene and rs659366 of the *UCP2* gene was performed using allele-specific amplification with real-time detection of results and by using TaqMan probes complementary to polymorphic DNA regions. The rs1801133 polymorphism of the *MTHFR* gene was studied using restriction fragment length polymorphism (RFLP) analysis. The study of resting energy expenditures and metabolic substrates (proteins, fats, carbohydrates) was carried out by indirect calorimetry using a stationary metabolic measuring device, namely “QuarkRMR” (“COSMED”, Italy).

### 2.4 Biochemical analysis

In patients with T2D, the levels of preprandial and postprandial glycemia as well as glycated hemoglobin (HbA1c) were determined by using a “One Touch® Ultra™” glucometer and a “KONELAB Prime 60i” biochemistry analyzer (“Thermo Scientific”, Finland). Serum biochemical parameters, including total cholesterol (TC), low-density lipoprotein cholesterol (LDL), high-density lipoprotein cholesterol, and triglycerides (TG), were also determined on the “KONELAB Prime 60i” biochemistry analyzer. Serum Hcy was determined by enzyme immunoassay using the “Axis Homocysteine EIA” (“AXIS-SHIELD”, Great Britain). Serum vitamin C content was determined photometrically using the Vitamin C kit (“Immundiagnostik AG”, Germany) and the content of vitamin B6 and folate in blood serum was measured microbiologically using “ID-Vit Vitamin B6” and “IDVit Folic acid” (“Immundiagnostik AG”, Germany). To determine the concentration of vitamin B12 and 25-hydroxyvitamin D in the blood serum, enzyme-linked immunosorbent assay (ELISA) was performed using “ID-Vit Vitamin B12” (“Immundiagnostik AG”, Germany) and “25-Hydroxy Vitamin D EIA” (“Immunodiagnostic systems”, UK).

### 2.5 Statistical analysis

The preliminary sample size was calculated using the WinPepi program to identify statistically significant differences in the studied parameters at the α-error level of 5% and with a statistical power of 80% [33].

Statistical data processing was performed using SPSS Statistics 21.0 software. The results were presented as mean values and standard error of the mean (mean ± SE). Statistical significance of differences in the samples was evaluated by the parametric Student t-test and one-way analysis of variance (ANOVA). The pairing relationship between two or more features was determined using the Spearman correlation analysis method. The significance level was considered significant at p < 0.05.

## 3 Results

Among the concomitant diseases, non-alcoholic fatty liver disease (78%), chronic superficial gastritis (62%), bilateral gonarthrosis (48%), chronic calculous cholecystitis (9%), and chronic pyelonephritis (6%) were detected most frequently. All chronic diseases were in the state of remission.

Regarding the *MTHFR* gene, it was found that more than half of the T2D patients were heterozygous, whereas genotype T/T was found in less than 8% of patients. In the *KCNJ11* gene, genotype C/C was found in less than 17% of patients, while heterozygous and homozygous genotypes regarding the T allele were almost equally distributed in case of the *UCP2* gene, and genotype C/C was found in 16% of patients.

A decrease in the rate of carbohydrates oxidation by 37.3 ± 5.8% and an increase in the rate of fat oxidation by 6.5 ± 0.9% were observed after assessing basal metabolism in 85% of patients with T2D and obesity. When studying the metabolic status in patients with variants of the *UCP2* gene, it was remarkable that the carbohydrate oxidation rate was significantly lower in the T than in the C allele carriers.

A study of carbohydrate metabolism in patients with T2D and obesity with variants of the *KCNJ11* gene revealed a significant difference in HbA1c and glycemia between the groups studied (**Figures 2 and 3**). No significant differences were seen in *MTHFR* gene variant carriers.

**Figure 1.**
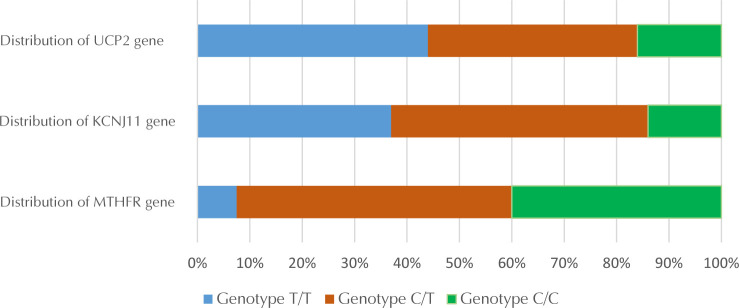
Distribution of variants of *MTHFR, KCNJ11*, and *UCP2*.

**Figure 2.**
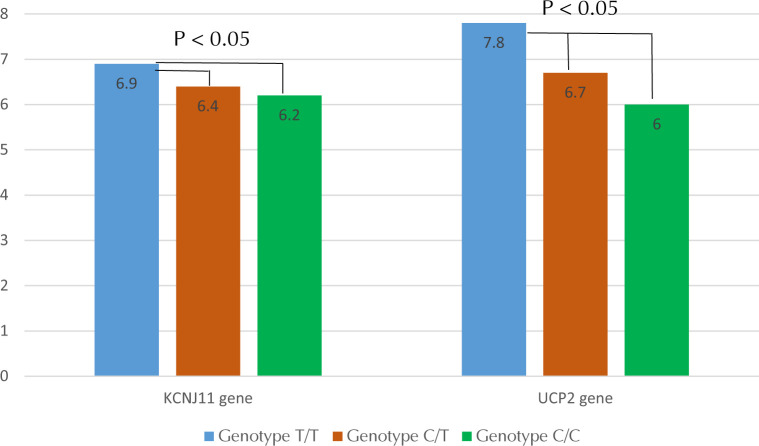
The level of glycated hemoglobin (%) in patients with type 2 diabetes and obesity in various polymorphic variants of *UCP2* and *KCNJ11*.

**Figure 3.**
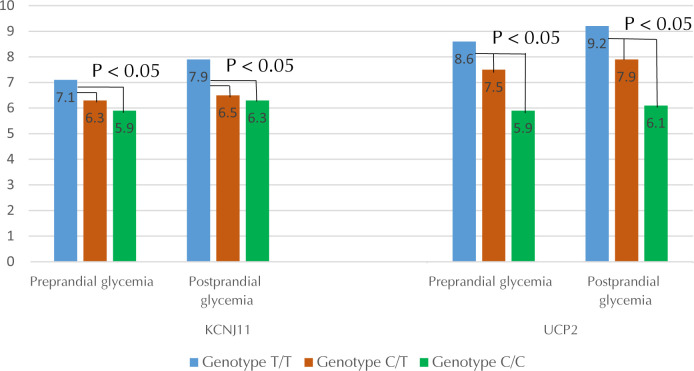
The level of glycemia (mmol/l) in patients with type 2 diabetes and obesity in various polymorphic variants of *UCP2* and *KCNJ11*.

The investigation of the carbohydrate metabolism in patients with variants of the *UCP2* gene showed that the level of HbA1c and glycemia in carriers of the T allele were significantly higher than in carriers of the C allele (**Figures 2 and 3**).

To verify confounding effects of the unfavorable variant of the *UCP2* gene on the results of the study into *KCNJ11*, carbohydrate metabolism in subjects with the T/T genotype was compared with that in subjects with the T/T variant in the *UCP2* gene and the C/C or C/T genotype in the *KCNJ11* gene. It was found that glycemia (8.2 mmol/l) and HbA1c levels (7.9%) in the first group were significantly higher than in the second group (6.3 mmol/l and 6.2% respectively), p < 0.05. The same was carried out in reverse order: the T/T variant of the *KCNJ11* gene remained constant in both groups. The first group included only carriers of the T/T variant of the *UCP2* gene, and the second group included the other two variants of this gene. In this case, the glycemic indicators (7.9 mmol/l) and HbA1c (6.8%) in the first group were higher than in the second group (7.5 mmol/l and 6.6%, respectively), but the differences were not statistically significant.

No significant differences could be detected in patients with different variants of *KCNJ11* and *UCP2* after evaluating lipid metabolism. When studying the effects of lipid metabolism in variants of the *MTHFR* gene, a significant increase in serum cholesterol, LDL cholesterol, and TG in blood serum could be observed in carriers of the T allele compared to those levels in carriers of the C allele.

The study of micronutrient levels in patients with T2D and obesity in variants of the *KCNJ11* gene again resulted in no significant differences between the groups. On the other hand, an intriguing result was discovered with variants of the *UCP2* gene, namely that the content of vitamin C in the blood serum of the carriers of the T/T genotype (8.2 ± 0.4 mg/l) was significantly lower than those with genotype C/T (10.5 ± 0.1 mg/l) and C/C (12.2 ± 0.4 mg/l), p < 0.05.

While determining Hcy as a risk factor for the development of vascular complications, moderate HHcy was observed in 42% of cases. Correlation analysis revealed a reliably positive relationship between Hcy, cholesterol, and blood serum TG levels (r = 0.358; r = 0.255, p < 0.05). When assessing Hcy in patients with variants of the *MTHFR* gene (Figure 5), it was noted that carriers of the C/T and T/T genotype had higher Hcy levels accompanied by lower vitamin B6, B12, and folate levels in the blood serum as compared to carriers of the C/C genotype. No differences were seen for carriers of variants in *KCNJ11* and *UCP2*.

**Figure 4.**
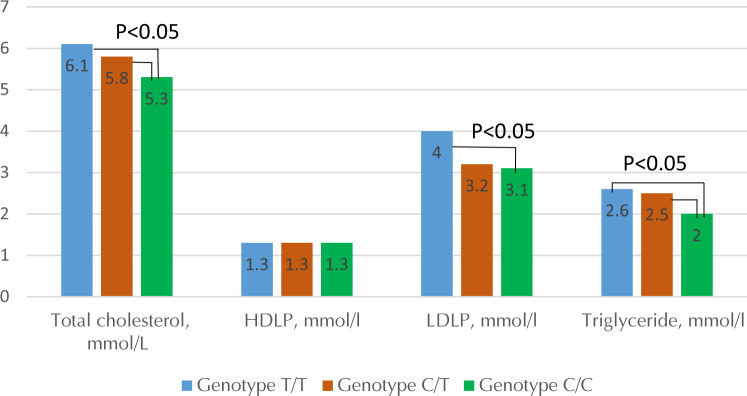
Lipid metabolism in patients with type 2 diabetes and obesity in various polymorphic variants of the *MTHFR* gene.

**Figure 5.**
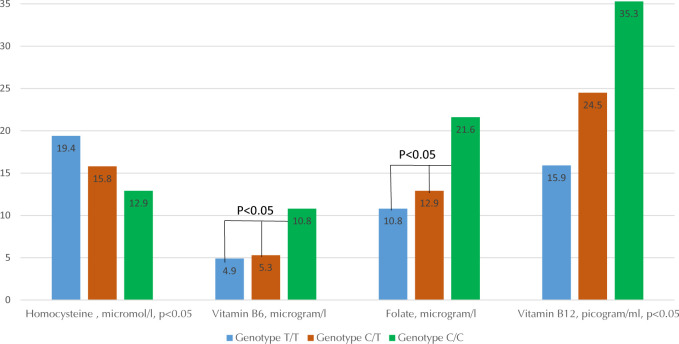
The content of homocysteine and different group B vitamins in the blood serum of patients with type 2 diabetes and obesity in various polymorphic variants of the *MTHFR* gene.

Correlation analysis revealed a negative relationship between Hcy and the content of vitamins B6, B12, and folate in the blood serum in all genotypes and in general (r = - 0.453; r = - 0.623; r = - 0.501, p < 0.05, respectively for all subjects).

**Figure 6.**
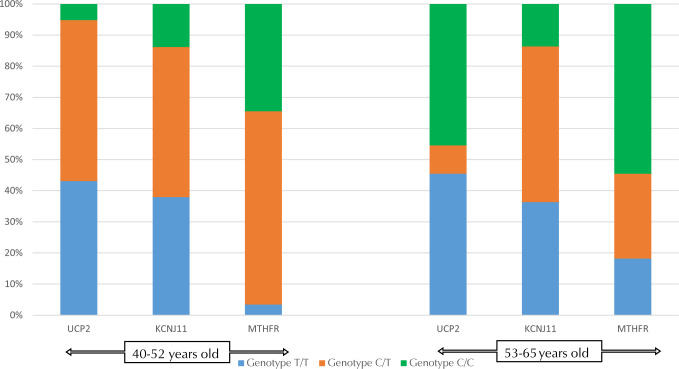
Distribution of *UCP2, KCNJ11*, and *MTHFR* genes by patient age.

**Figure 7.**
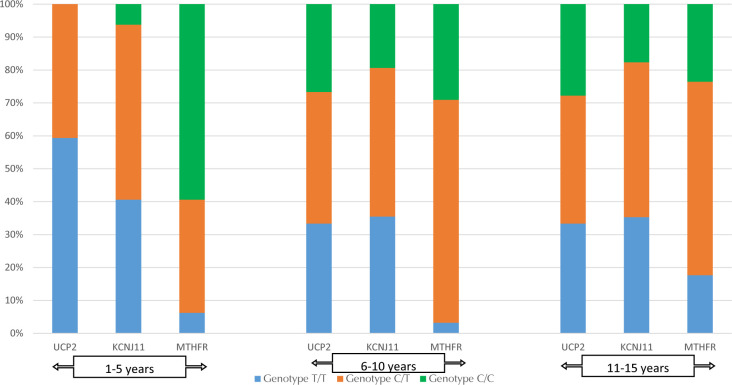
Distribution of *UCP2, KCNJ11*, and *MTHFR* genes by duration of diabetes.

To reduce the risk of bias caused by the uneven distribution of genotypes in the study group, the influence of the prevalence of an unfavorable genotype in older patients or in patients with a long history of diabetes, the distribution of the genotypes in subgroups was assessed by age (58 people in the 40-52 year age group vs. 22 people in the 53-65 year age group) and by the duration of diabetes (32 patients: 1-5 years vs. 31 people: 6-10 years vs. 17 people: 11-15 years). There was no statistically significant difference between the groups in either case (**Figures 6 and 7**).

## 4 Discussion

### 4.1 MTHFR gene

Genotype C/C of *MTHFR* seemed to be more favorable for lipid metabolism as it resulted in lower levels of LDL, triglycerides, and total cholesterol. On the other hand, the T/T genotype of the same gene had exactly opposite effects, i.e. higher triglyceride, LDL, and total cholesterol. Similarly, the C/C genotype of *MTHFR* resulted in lower Hcy and a higher level of vitamin B6, B9, and B12, whereas T/T genotype carriers had increased Hcy and decreased vitamin B6, B9, and B12 levels. Consequently, patients with the C/C genotype may have a reduced risk of developing cardiovascular diseases compared with patients with the T/T genotype. Therefore, patients with the T/T genotype are recommended to monitor vitamin and Hcy status regularly.

### 4.2 KCNJ11 gene

The C/C genotype of the *KCNJ11* gene was most favorable regarding glycated hemoglobin. The C/T genotype was not quite as favorable as the C/C, and the T/T genotype was associated with the highest level of glycated hemoglobin. Similarly, the level of pre- and postprandial glycemia in people with the C/C genotype seemed to be the lowest in comparison with C/T and C/T genotypes. The T/T genotype was related to the highest levels of pre- and postprandial glycemia. However, the differences obtained were partially explained by the carrier of an unfavorable variant of the *UCP2* gene, since comparison of the variants of the *KCNJ11* gene in carriers of only one variant of the *UCP2* gene (T/T) showed that the T/T genotype for the *KCNJ11* gene remained the least favorable, but the differences were not statistically significant. As the higher level of glycated hemoglobin, pre- and postprandial glycemia are indicators of T2D and obesity, the C/C genotype of the *KCNJ11* gene seems to have the best prognosis.

### 4.3 UCP2 gene

The effects of different variations of the *UCP2* gene were similar to the different variants of the *KCNJ11* gene. The C/C genotype appeared to maintain the lowest level of glycated hemoglobin and the T/T genotype the highest. The C/T genotype was associated with an intermediate level of glycated hemoglobin. Likewise, pre- and postprandial glycemia were also lowest for C/C genotype, highest for T/T genotype, and intermediate level for the C/T genotype. The lower level of glycated hemoglobin and pre- and postprandial glycemia was associated with lower risk of T2D, obesity, and cardiovascular disease. Similarly, it can also decrease complications during diseases progression. Therefore, C/C was the most favorable genotype.

### 4.4 Limitations of the study

This study had several limitations. Firstly, the majority of patients needed to be excluded from the study as they required a change in therapy during the study period. Even a small change in the dosage of metformin can significantly affect carbohydrate metabolism and the levels of magnesium and vitamins B1, B9, B12, and D [34]. Therefore, the number of subjects completing the study was only 80. Heterogeneity in the distribution of genotypes led to a situation where some groups included less than 10 subjects (e.g. the number of the T/T variant of the *MTHFR* gene carriers). When dividing the total sample by age or duration of diabetes, the groups of carriers of certain genotypes became even smaller.

Furthermore, the results of the study cannot be confidently extrapolated to the entire population as the effects have been described for women only and the distribution of genotypes in the study group does not necessarily match the distribution of these genotypes in the population. Another source of bias could be that the carriers of the most favorable genotypes may have a lower risk of developing diabetes, preventing them from being included in the study in the first place. This could ultimately increase the proportion of unfavorable genotypes in the study.

An additional source of bias could be that patients’ diets were not standardized. The levels of glycosylated hemoglobin, lipids, and vitamins might be significantly influenced by the diet that the subjects followed prior to the study. At the same time, the groups were absolutely homogeneous in terms of sex composition and pharmacotherapy, which reduced the risk of bias.

To verify the results of this study, further research is needed in a larger sample. Also, a global population study is necessary to assess the distribution of these genotypes in the population as a whole, also among healthy people. Similarly, the same research can be repeated in patients with different durations of diabetes to determine the role of these genes in the outcome of the disease. Finally, a similar study in the male sex could also provide further information.

## 5 Conclusions

The genotype of the patients affects the prognosis of the disease. Therefore, genotyping of such patients can help in more qualitative selection of treatment and assessment of future risk.

Certain genotypes may require specific attention to their physiological features. Carriers of the defective MTHFR gene should be monitored regarding their Hcy concentration and indicators of its metabolism such as vitamins B6, B9, and B12. Their vascular risk is elevated by higher levels of TC, LDL, and TG. Carriers of the T/T variant of the UCP2 and KCNJ11 gene show poor carbohydrate metabolism, and probably are more resistant to antihyperglycemic therapy. They may have a higher risk of developing T2D. However, more research is needed to verify these findings.

## Abbreviations

ELISA enzyme-linked immunosorbent assay

HbA1c glycated hemoglobin

Hcy homocysteine HHcy hyperhomocysteinemia

KCNJ11 potassium inwardly rectifying channel subfamily J member 11

LDL low-density lipoprotein MTHFR methylenetetrahydrofolate reductase

RFLP restriction fragment length polymorphism

T2D type 2 diabetes

TC total cholesterol

TG triglyceride

UCP2 uncoupling protein 2

WHO World Health Organization
